# COVID-19 Detection by Optimizing Deep Residual Features with Improved Clustering-Based Golden Ratio Optimizer

**DOI:** 10.3390/diagnostics11020315

**Published:** 2021-02-15

**Authors:** Soham Chattopadhyay, Arijit Dey, Pawan Kumar Singh, Zong Woo Geem, Ram Sarkar

**Affiliations:** 1 Department of Electrical Engineering, Jadavpur University, Kolkata 700032, India; chattopadhyaysoham99@gmail.com; 2 Department of Computer Science and Engineering, Maulana Abul Kalam Azad University of Technology, Simhat, Haringhata, Nadia 741249, India; arijjitdey3413@gmail.com; 3 Department of Information Technology, Jadavpur University, Kolkata 700106, India; pksingh.it@jadavpuruniversity.in; 4 College of IT Convergence, Gachon University, 1342 Seongnam Daero, Seongnam 13120, Korea; 5 Department of Computer Science and Engineering, Jadavpur University, Kolkata 700032, India; ram.sarkar@jadavpuruniversity.in

**Keywords:** COVID-19 detection, CGRO algorithm, deep features, meta-heuristic, feature selection, CT-scan, chest X-ray

## Abstract

The COVID-19 virus is spreading across the world very rapidly. The World Health Organization (WHO) declared it a global pandemic on 11 March 2020. Early detection of this virus is necessary because of the unavailability of any specific drug. The researchers have developed different techniques for COVID-19 detection, but only a few of them have achieved satisfactory results. There are three ways for COVID-19 detection to date, those are real-time reverse transcription-polymerize chain reaction (RT-PCR), Computed Tomography (CT), and X-ray plays. In this work, we have proposed a less expensive computational model for automatic COVID-19 detection from Chest X-ray and CT-scan images. Our paper has a two-fold contribution. Initially, we have extracted deep features from the image dataset and then introduced a completely novel meta-heuristic feature selection approach, named Clustering-based Golden Ratio Optimizer (CGRO). The model has been implemented on three publicly available datasets, namely the COVID CT-dataset, SARS-Cov-2 dataset, and Chest X-Ray dataset, and attained state-of-the-art accuracies of 99.31%, 98.65%, and 99.44%, respectively.

## 1. Introduction

The Coronavirus was first noticed in Wuhan city, China. Other than Antarctica, almost every continent has been more or less affected. Scientists predict that the virus originated from zoonotic natured animals. However, the origin of this virus is not yet been discovered [1]. The first infected person was from Wuhan market in Hubei province and it eventually spread across the globe [2]. This virus has evolved itself in the recent decades, in 2002 it was known as Severe Acute Respiratory Syndrome Coronavirus (SARS-CoV) and in 2012 it was known as the Middle East Respiratory Syndrome Coronavirus. However, in 2019, the World Health Organization (WHO) that declared an unknown etiology had been detected in the city of Wuhan, which is a novel coronavirus, named 2019 coronavirus (2019-nCoV), and that can cause severe pneumonia [3]. In 2020, the International Committee on Taxonomy of Virus (ICTV) announced the 2019 coronavirus as SARS-Cov-2, and the disease as Coronavirus disease 2019 [4,5].

**Figure 1:**
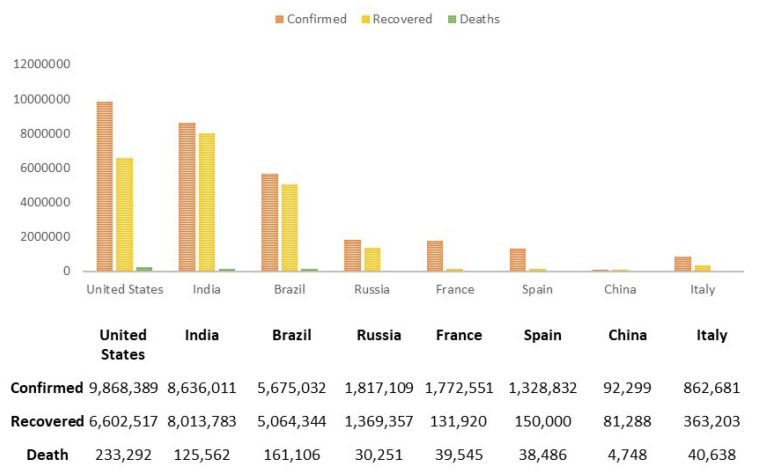
Statistics of confirmed, recovered and death cases of COVID-19 in some countries until 11th November [7].

Globally, 49,106,931 people are affected, among them 1,239,157 people unfortunately lost their battle as of 11 November 2020 [6]. The most affected country, to date, is the USA, having a total of 9.3 million confirmed cases. By mid-March, Italy had the highest amount of deaths [7]. India is in second place according to the number of confirmed cases. However, China has managed to avoid the list of the top 10 most affected countries of COVID-19. Figure 1 shows the detailed statistics of COVID-19 in some countries.

**Figure 2:**
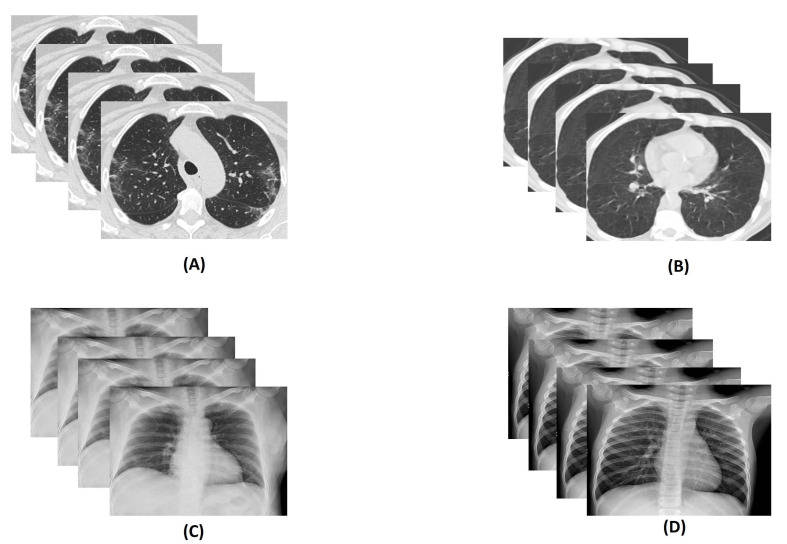
Illustration of sample: (**A**) COVID Computed Tomography (CT) scan images, (**B**) non-COVID CT scan images, (**C**) COVID X-ray images, and (**D**) non-COVID X-ray images.

Usually, the most settled way for COVID-19 detection is real-time reverse transcription-polymerize chain reaction (RT-PCR). However, RT-PCR has a low diagnosis accuracy, 60–70%. Many times it is evident that even after getting negative results symptoms can be detected by radiological images of patients [8]. Computed Tomography (CT) and X-ray play an important role in detecting life-threatening diseases [9]. Usually, RT-PCR takes many hours, even a day. That is the reason CT scan and X-ray have been used as a sensitive and fast method for diagnosis COVID-19 [10]. However, the findings in lungs because of COVID-19 is visible after two days [11], and the most significant result is observed after 10 days [12]. Moreover, the COVID-19 virus affects the lungs of a suspected person and, eventually, the lung becomes puffed up. An experiment says that shadowy patches can be shown in the CT scan and X-ray image of the chest of an infected person; this phenomenon is known as Ground Glass Opacity [13]. Figure 2 shows some samples of the COVID and non-COVID CT scan and X-ray images. Additionally, this virus spreads much faster than it’s prediction and detection rate due to its communicable nature. The symptoms of COVID-19 is quite similar to one chronic disease, pneumonia. The lungs also become inflamed in this case, and it is also life-threatening but not as fatal as COVID-19.

In recent times, many computer aided detection (CAD) systems [14] have been proposed that help to detect different chronic diseases accurately, such as lung cancer [15], breast cancer [16], skin cancer [17], and brain cancer [18].

**Figure 3:**
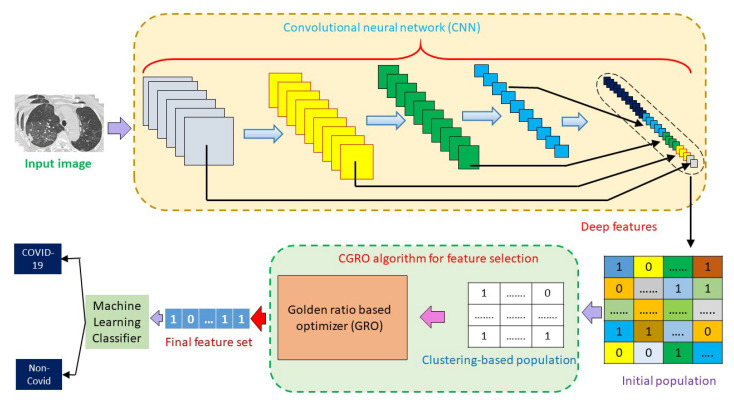
Workflow of the proposed Cluster-based Golden Ratio based Optimizer (CGRO) based feature selection approach for COVID-19 detection.

However, we have implemented an ensemble of deep learning and machine learning (ML) techniques for the detection of COVID-19 cases. It is known that deep learning models can learn relevant features by themselves. On the other hand, to use purely ML techniques, various features are extracted from the input data, by manual effort, which, many times, cannot provide the state-of-the-art (SOTA) results, and some chances always remain for extracting redundant features and missing out some of the relevant features. Therefore, the features of a deep learning model learns more prolific and compact than that of manually extracted traditional features. In contrast, the last or the classification layer of any deep learning model is not much optimized like any ML classifier. Therefore, the classifying efficiency of that particular deep layer is less than that of ML-based classifiers. Keeping these facts in mind, in the current work, we have developed an ensemble framework, which includes deep features from pre-trained Convolutional Neural Networks (CNNs) and a wrapper based optimization technique for feature selection (FS) and classification. Figure 3 illustrates the complete workflow of our proposed approach for COVID-19 detection. The contributions of the present work are briefly described below:

- We have extracted deep features from different layers of pre-trained ResNet18, which is trained for 30 epochs on our datasets and those are concatenated in order to obtain the final feature set.
- A new FS method, called Cluster-based Golden Ratio based Optimizer (CGRO), is introduced, which includes clustering-based population generation to avoid premature convergence of the algorithm.
- The model is evaluated using three SOTA classifiers, namely Support Vector Machine (SVM), K-Nearest Neighbors (KNN), and Extreme learning machine (ELM), on three standard COVID-19 datasets, namely the Covid-CT dataset [19], SARS-Cov-2 CT-Scan dataset [20], and Chest X-Ray dataset [21]. The first two datasets are CT-Scan image-based datasets and the last one is based on the chest X-Ray dataset. On all three datasets, the proposed approach achieves SOTA results, with a good margin of difference from recently developed models.
- We have also compared the performance of the CGRO algorithm with some popularly used FS based optimization algorithms on all three datasets, which are reported in the results and discussion section.

## 2. Related Work

In recent times, many ML, as well as deep learning-based approaches, are introduced for COVID-19 detection from both the X-ray and CT scan images. X-ray and CT scan images are both the best way to analyze COVID-19 data. X-rays have been preferred over the chest CT scans due to less ionizing radiations and portability [22]. However, there are also few limitations of chest X-rays over CT scans, such as X-ray detecting information of the lung according to the shape, size, the structure of lungs, whereas CT scan images give an informative architecture of air sacs. However, in our present work, we have implemented our model taking inputs from both the datasets (i.e., CT scans and X-rays) for a comprehensive study.

The initial constraint in this field of research is the lack of data. For this reason, Wang et al. [7] proposed a data augmentation technique, Auxiliary Classifier Generative Adversarial Network (ACGAN), which helps to manage a sufficient number of radiographic images within a limited period. It also helps to improve the performance of Convolutional Neural Network (CNN). Wang et al. [23] implemented a deep CNN to identify COVID-19 positive from the X-ray images. They have trained the model with 13,975 chest X-ray images and produced a classification accuracy of 98.9%. However, Hemdan et al. [24] introduced the COVIDX-Net model that can detect COVID-19 infected from X-ray images. COVIDX-Net is trained with 50 normal and 25 COVID images, and it scored a classification accuracy of 91%. Md. ZabirulIslam [25] proposed a new technique for diagnosing COVID-19 automatically from the X-ray images using a combined deep CNN-LSTM network. The model is trained with 4575 X-ray images, including 1525 images of COVID-19, which produced an accuracy of 99.4%. B. Abraham et al. [26] have investigated the effectiveness of multi-CNN by using the combination of several pre-trained CNNs for COVID-19 detection. They extracted deep features from multi-CNN and carried the processing ahead using the correlation-based FS (CFS) technique. They trained the model on 453 COVID-19 images and 497 non-COVID images, and obtained an accuracy of 91.16%. Again, K.H. Shibly et al. [27] proposed a technique, named faster R-CNN, to detect COVID-19 from X-ray images. They have implemented their model on two publicly available datasets, one is a customized dataset and another one is COVIDx, and obtained an accuracy of 97.36% and 97.65%. Wang and Wong [28] proposed a unique deep learning technique, named COVID-Net, which obtained 92.4% classification accuracy. Furthermore, Ioannis et al. [29] implemented a deep learning model while using 224 confirmed COVID images and achieved an accuracy of 98.75%.

In our work, we have implemented our model on three publicly available datasets, namely SARS-Cov-2, Muhammed Talo 2 class, and COVID-CT datasets. There are only a few works reported on these datasets, which are described here. Loey et al. [30] extracted deep features from the COVID-CT dataset and then they have implemented augmentation using CGAN. They obtained a classification accuracy of 82.91%. However, Jhao et al. [19] used pre-trained CNN for classification and achieved an accuracy of 89.1% on the same dataset. Furthermore, Saeedi et al. [31] extracted deep features using DeepNet121 of the COVID-CT dataset and used Nu-SVM for the classification purpose. They scored an overall accuracy of 90.61%. Whereas, Shaban el al. [32] proposed a new approach, hybrid feature selection Methodology (HFSM) and achieved an impressive classification accuracy of 96% on the COVID-CT dataset while using enhanced K-Nearest Neighbor (EKNN) classifier.

Jaiswal et al. [33] have used DenseNet 201 on the SARS-Cov-2 dataset and obtained an accuracy of 96.25%. However, Soares et al. [20] implemented xDNN for classification and achieved an accuracy of 97.38% on the same dataset. Panwar et al. [34] implemented Gradient-weighted Class Activation Mapping (Grad-CAM) on the same dataset and scored an accuracy of 95.61%. Again, Ozturk et al. [21] implemented the DarkCovidNet model that produced an accuracy of 97.08% on the Chest X-ray dataset that was proposed by Muhammad Talo. Further, Abdulrahaman et al. [35] introduced a deep Belief network to attain an accuracy of 90% on the same dataset.

Moreover, in our literature, we have used a completely novel FS technique to neglect redundant features from the extracted deep feature set. Meta-heuristic [36] approaches are quite popular for managing this task. In recent times, many optimization techniques have been introduced, and it has been an area of interest among the research fraternity. However, many optimization algorithms are already available for different tasks, but there is always an intermediate need to develop optimization algorithms for a specific task. Researchers have found that a single optimization algorithm might fail to deal with every problem [37]. That is why researchers have developed different optimization algorithms in different domains to deal with redundant features and it can enhance both exploration and exploitation capability. Some famous and most recent hybrid FS algorithms proposed during recent times are, as follows: Binary Bat Algorithm with Late Acceptance Hill-Climbing (BBA-LAHC) [38], hybridization of Mayfly algorithm (MA), and HS, named as the MA-HS algorithm [39], cooperative Genetic Algorithm (CGA) [40], hybridization of GA with PSO and Ant Colony Optimization (ACO) algorithm [41], hybrid golden ratio optimization and equilibrium (GREO)  [42], and clustering-based equilibrium and ant colony optimization (EOAS) [42].

## 3. Motivation

In recent times, many COVID-19 infected patients are asymptomatic, which might increase the transmission without any symptoms [43]. RT-PCR is the most common method of COVID-19 detection. It can be done by taking sputum or blood samples of infected patients [44]. However, it takes a few hours or even a day to get the result [45]. On the other hand, using biological image processing, our model can automaticallydetect COVID-19. Most of the COVID-19 works are particularly using deep learning models. However, such models sometimes generate many redundant and non-informative features. Hence, we aim to find out an optimal feature subset from the deep features that were extracted from the COVID-19 affected CT scan and X-ray images. Hybrid FS models are quite famous among the researchers, as it focuses on both exploration and exploitation. There are a lot of hybrid FS models available in the literature, such as Electrical Harmony based hybrid meta-heuristic (EHHM) [46], Hybrid of Harmony Search Algorithm and Ring Theory-Based Evolutionary Algorithm [47], Mayfly in Harmony [39], and Binary Social Mimic Optimization Algorithm with X-Shaped Transfer Function [48]. Successful applications of FS algorithms in various domains have motivated us to propose a new FS algorithm for COVID-19 detection.

## 4. Materials and Methods

In this section, the proposed workflow for COVID-19 detection has been discussed in a sequential manner. The methodology is divided into three subsections: Section 4.1 Dataset Description, Section 4.2 Deep Residual Feature Extraction, and Section 4.3 Feature Selection.

### 4.1. Dataset Description

In this study, we have evaluated our model on three benchmark datasets, which are briefly described below.

#### 4.1.1. COVID-CT Dataset

The Covid-CT dataset was proposed by Jhao et al. [19]. As the name suggests, this dataset consists of chest CT-Scan images with 349 confirm COVID-19 cases and 397 healthy cases. In this research framework, all of the images are resized to 224 × 224 × 3, and they are normalized to increase the robustness of usage in the domain of various deep learning frameworks. During the training process of deep neural networks, as the dataset is very small, the images are augmented by rotation of $50^{\circ }$, slant-angle of $0.5^{\circ }$, and by enabling horizontal and vertical flipping. Here, 597 images are taken for training the model and the remaining 74 images are taken as the testing data.

#### 4.1.2. SARS-Cov-2 Dataset

Sores et al. proposed the SARS-Cov-2 CT Scan dataset [20]. This dataset contains 2492 chest CT scan images, 1262 of which are COVID-19 positive, and the remaining 1230 images are of healthy subjects. Similar to the previous dataset, here also the images are resized to 224 × 224 × 3, and, during training, data augmentation is applied with $25^{\circ }$ of rotation and horizontal flip. Here, 1994 images are taken for training the model and the remaining 249 images are taken as testing data.

#### 4.1.3. X-ray Dataset Proposed by Muhammed Talo

The final dataset is a chest X-ray dataset [21], which a fusion of two datasets. One of which is an X-ray dataset from Kaggle and the other one is a dataset consists of 125 X-ray images, collected from various open sources by JP Cohen [49]. Here, 800 images are taken for training the model and the remaining 100 images are taken as testing data.

### 4.2. Deep Residual Feature Extraction

In this present framework, we have extracted deep residual features instead of traditional hand-crafted features. There are many traditional feature extraction techniques, like Gabor [50], Haralick [51], Tamura [52], etc., which provide hand-crafted features. Sometimes researchers need exhaustive experimentation to decide which features can be of the most relevance for the said classification task. Even after such experimentation, there often remains some redundant features as well as missing significant features in the manually prepared feature set.

On the other hand, CNNs learn through the backpropagation technique, mostly such models only learn relevant features. However, some of the features may be less informative and they do not contribute more toward classification. Therefore, we have applied a meta-heuristic based FS algorithm to further optimize the features extracted from a deep CNN model. Here, we have extracted features from different layers of ResNet18. We have also extracted features from other popularly used CNNs, and the comparative results help us to finalize the CNN model for feature extraction.

For feature extraction purposes, we have implemented traditional transfer learning techniques with a pre-trained ResNet18 model. Deep CNNs, like ResNet18, have many layers in them. The initial layers of any CNN mainly learn common features, which are responsible for defining the geometrical abstractions of the image. These higher dimensional features are considerably sensitive towards noises, disorders, and repressions. Whereas, the deeper layers learn the shape of the images specific to the classification task at hand. These are comparatively more robust and viable in the cases of clutters and occlusions. Besides, local features contain more relevant information regarding the patterns, such as edges, contours, textures, etc., of the input images. To utilize the combined information of both local and global features, we extract features maps from different layers of ResNet18 and concatenated them to form the final feature set. To accomplish the task, we firstly fine-tune the weights and biases of pre-trained ResNet18 architecture by training the network for 30 epochs and save the model with minimum validation loss. Thereafter, all of the images in the dataset, including train and test images, are passed into the network, and feature maps of different layers, such as layer1, layer2, layer3, and layer4, are extracted via an adaptive average pool layer with kernel size equals to 1 × 1. This average pool layer normalizes the feature dimension (height and width) to 1 only. These higher dimensional feature maps are further flattened and concatenated and the final feature set is achieved. Table 1 displays the feature maps and detailed feature extraction from different layers.

**Figure 4:**
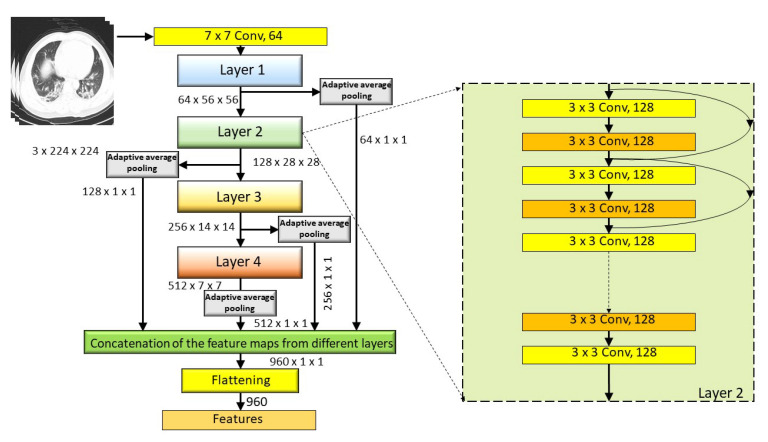
Pictorial representation of our proposed approach of deep feature extraction from fine tuned ResNet18 network.

This combined feature set, which consists of 960 features (see Figure 4 for more detail), is further optimized using our proposed CGRO based FS algorithm for generating the final feature set used for the classification task.

### 4.3. Feature Selection Model

In this section, a completely new approach to feature selection has been discussed to reduce the redundancy of the deep residual features. For this purpose, we have proposed a completely new approach for the FS task, named CGRO.

#### 4.3.1. Golden Ratio Optimization Algorithm

Every element in nature has its specific shape and size. They follow a similar pattern and every physical entity has its fixed proportion, called the golden ratio (GRO) [53]. Fibonacci first proposed the idea of the golden ratio. He introduced some series of numbers, which are exactly the sum of their previous two numbers and the ratio of two consecutive numbers is exactly 1.618. This ratio is known as the golden ratio. The equation that describes this property is given below.

(1) $$P\left(n\right)=F\;.\frac{\left(\;\epsilon^{n}-\left(1-\;\epsilon^{-n}\right)\right)}{\sqrt{5}}\;\;\;\;where\;F\;=\;1.618$$

This optimization technique deals with the vectors and the direction of the vector to obtain the best solution. Initially, the mean of the population is calculated and, after that, the fitness is calculated. Based on the calculated fitness, the best and worst fitness are assigned. In the next stage, one random population is generated and the impact on the population for the movement of the best and worst solution vector is calculated. Subsequently, the optimization process will go a step forward towards optimization. The process will go on iteratively. The pseudo-code, as described in Algorithm 1, shows how the GRO algorithm works.

(2) $$P_{best}\;>\;P_{medium}>\;P_{worst}$$

(3) $$Z_{t\;}=\;Z_{medium\;}-\;Z_{worst\;}$$

The above equation gives the information about the modulus value of the movement and the corresponding direction, in search of the global minimum. Fibonacci’s formula is used to perform the global and local search operation. Updating the solution is the next step. The equation that represents the random movement is given below.

(4) $$Z_{new\;\;}=\left(1-P_{t\;}\right)Z_{best\;}+rand.Z_{t\;}.P_{t\;}$$

**Algorithm 1 Pseudo code for GRO algorithm****Input**: Whole feature set, no-of-population, max-iter **Output**: Final solution
1:Population initialization k=1,2,3,…,n2:Fitness calculation3:**while** Convergence criterion is not satisfied **do**4:   Obtain Zav, the mean value of all possible solutions5:   Set the worst fitness as $Z_{worst}$6:   **if** FITNESS($Z_{avg}$) < FITNESS($Z_{worst}$) **then**7:      swap($Z_{avg}$,$Z_{worst}$)8:   **end if**9:   **for** J=1, *…*, particle number **do**10:      Random population generation $Z_{k}$11:      Compare $Z_{I},Z_{j},Z_{avg}$ and assign best fitness value in the $Z_{best}$ and worst value in the $Z_{worst}$12:      Evaluate on **eq 1**.13:      Check the constraints14:      $Z_{t}$ = $Z_{median}-Z_{worst}$15:   **end for**16:   **for** j=1, *…*, number of particles **do**17:      **for** k=1, *…*, number of variables **do**18:         Update the solution $Z_{new}=\left(1-P_{t}\right)Z_{best}+rand.Z_{t}.P_{t}$19:         Check the constraints20:      **end for**21:   **end for**22:**end while**23:**Output:** Final Solution

Now, the new solution is updated and, if the boundary condition is satisfied, then the new solution will be replaced with the previous one.

#### 4.3.2. Clustering-Based Population Selection

The GRO algorithm usually has a high convergence rate. Accordingly, there is a chance that the GRO algorithm may get stuck at the local optima, which results in non-desired solution, i.e., the algorithm fails to reach the global optima. In this context, it is to be noted that, if the initial population is generated randomly, then the candidate solutions may have less diversity and their exploration abilities can be severely affected. Therefore, at the starting of randomized initialization, significantly distributed candidate solutions are considered to address this premature convergence of GRO. In doing so, we apply a clustering-based population selection concept on the deep features obtained previously [54].

For the clustering-based population, initially, **n** randomly generated candidate solutions are selected as the initial population. However, a random number is generated as hyper-parameter to address the number of cluster centers is required, which is less than the number of candidate solutions in the population. In addition, **c** clusters are initiated by considering another randomly generated **c** cluster centres, each being assigned to a single cluster. It is to be noted that **c** should always be less than **p** and greater than 1. Now, a similarity function is defined, which is given by Equation (6).

(5) $$S=\alpha \times d_{H}+\left(1-\alpha \right)\times a_{D}$$

Using Equation (6), the similarity is calculated between each candidate solution to the cluster centers. In the equation, **$d_{H}$** and **$a_{D}$** are the hamming distance and the difference in classification accuracy between the solution vector of the population and the cluster center, respectively. These terms are added via weights **α** and **1−α**. The term, **$d_{H}$**, represents the ease of bringing a particle to a certain cluster center, whereas the second term gives the information regarding the closure proximity between the classification abilities of the two particles. Now, a particle is assigned to that cluster for which the cluster center gives maximum similarity with the candidate solution. After this process, each cluster gets some candidate solutions based on the calculated similarity values.

**Figure 5:**
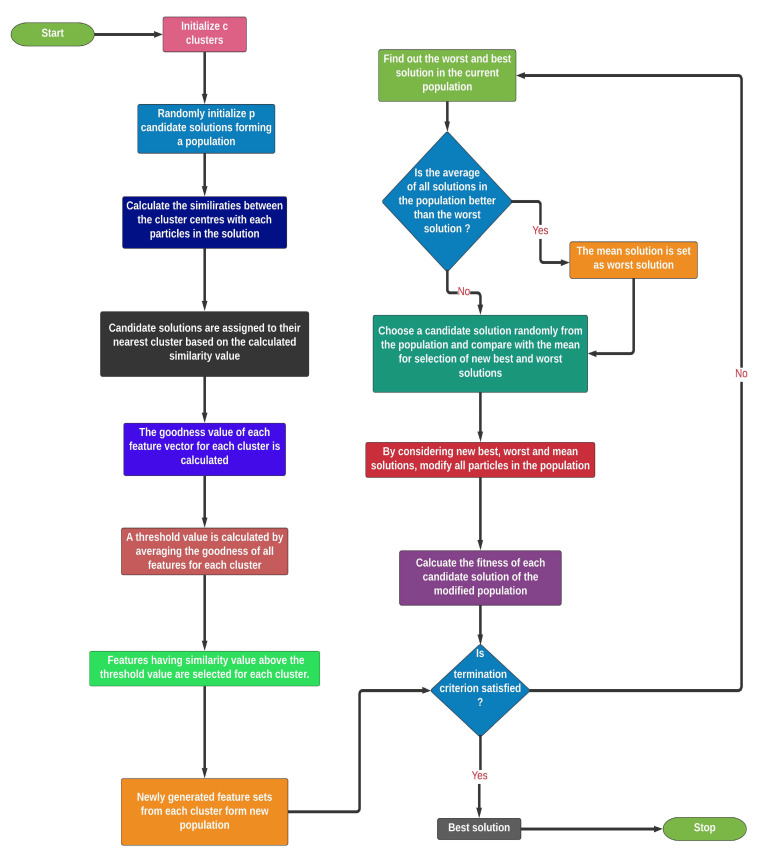
Flowchart of our proposed CGRO based FS algorithm.

Suppose that the **$C^{th}$** cluster is assigned with M particles. Now, in cluster C, when considering m particles, a goodness measure is calculated for each feature vector. The mathematical expression of the goodness value of **$k^{th}$** feature vector is given by Equation (7).

(6) $$G_{k}^{C}=\sum_{m=1}^{M}r_{mk}^{C}\times A_{m}^{C}$$

where, **$G_{k}^{C}$** is the goodness of **$k^{th}$** feature vector of **$C^{th}$** cluster. The expression of goodness function is based on two main terms, the position of the particle in **$C^{th}$** cluster (**$r_{mk}^{C}$**) and the classification accuracy **$\left(A_{m}^{C}\right)$** of each particle in the cluster. The cumulative sum over all the particles in the cluster of the multiplication of the aforementioned two terms gives the goodness measure of **$m^{th}$** feature vector. In the next stage of final solution vector generation from a cluster, a feature vector is selected if the goodness value of the feature vector is greater than the mean goodness measure of all feature vectors. Thus, at the end of this clustering process, we have c standard candidate solutions out of n randomly generated population. Figure 5 presents the flowchart of our proposed CGRO based FS algorithm.

### 4.4. Overview of the Classifiers Used

For the calculation of fitness function, we have chosen three different SOTA classifiers, such as SVM, KNN, and ELM. Brief descriptions of these classifiers are given below.

#### 4.4.1. SVM

SVM [55] is a popularly used supervised learning algorithm, which is also used for classification and regression problems. SVM is a linear classifier, but, while using kernel-trick, it can also achieve efficient performance for non-linear classifications. SVM projects the training sample data points to a higher dimensional space and draws several hyperplanes that separate different classes into the hyperplane. SVM makes sure that the hyperplanes are kept at the furthest distance from the elements of each class. Now, while testing, the SVM algorithm decides at which side of a hyperplane a single test data point should be put, and that is the determined class of that point. Several kernel functions are used to project the training points into the higher dimensional spaces. Some of the popularly used kernel functions are rbf,Gaussian,polynomial, and so on. Among these kernel functions, rbf performs superior to others while the feature space is large.

#### 4.4.2. KNN

KNN [56] is another heavily used ML algorithm, which is used in pattern recognition tasks. The algorithm of KNN is of non-parametric type and applied in both classification and regression tasks. Here, the input consists of K closest samples of training out of the entire feature space. For classification, this lazy learning algorithm assigns the output as a class membership. Classification is done by a plurality vote of nearest neighbors and the data point is classified to the class that is chosen by a majority of the neighbors. This is how, by a voting system, the KNN classifier works.

#### 4.4.3. ELM

ELM [57] is a feed-forward neural network, with some layers of hidden nodes, which are usually assigned randomly. The output weights and biases of different hidden nodes are upgraded in a single step, which is the learning of a linear model. For the ELM classifier, no back-propagation algorithm occurs, which results in faster learning than usual neural networks and achieves good performance. ELM is mostly used with a single hidden node, but it also has flexible architecture. ELM can also be used with RBF networks, sigmoid-based complex neural networks, wavelet transform, and fuzzy inference networks. For ELM based networks, the hidden node can also be a single neuron or a basis function or subnetwork.

## 5. Results and Discussion

A novel FS based approach for COVID-19 detection is reported in this paper. The proposed framework of optimizing deep features are evaluated on three recently proposed COVID-19 detection datasets, namely the COVID-CT Dataset, as proposed by Zhao et al. [19], SARS-Cov-2 CT scan dataset, which was proposed by Soares et al. [20], and the chest X-Ray dataset, as proposed by Muhammed Talo [21]. For the evaluation of our model, we have relied upon some standard measures used for statistical evaluations, such as Accuracy, Recall, Precision, and F1 Score. These evaluation metrics are dependent on some primary measures, which are True Positive (TP), True Negative (TN), False Positive (FP), and False Negative (FN). These evaluation metrics, in terms of the elementary measures, are given by the following equations:

- **Accuracy:**
  (7) $$\frac{TP+TN}{TP+TN+FP+FN}$$
- **Precision:**
  (8) $$\frac{TP}{FP+TP}$$
- **Recall:**
  (9) $$\frac{TP}{TP+FN}$$
- **F1 Score:**
  (10) $$\frac{TP}{TP+\frac{1}{2}\left(FP+FN\right)}$$

In this current study, we have trained our pre-trained CNN model for 30 epochs, which basically fine-tune the pre-trained weights and then fix the fine-tuned weights and extracted features from different layers of it.

### 5.1. Deep Feature Extractors

In the previous section it is mentioned that we have chosen deep features instead of the traditional feature engineering approach for our current framework of FS and classification. For this purpose, we have performed exhaustive experimentation to select an appropriate CNN model for feature extraction. We have considered several pre-trained deep learning models, like GoogleNet [58], ResNet18 [59], VGG19, VGG16 [60], and ResNet152 [61], for deep feature extraction. We have also extracted feature maps from different layers of ResNet18 and concatenated them after adaptive average pooling and flattening, which gives a feature vector of 960 features. Table 2, Table 3, Table 4 and Table 5 display the results obtained from this comparative study of different deep learning models for the chosen datasets .

It is observed from the results reported in the tables that the proposed approach of deep feature extraction achieves the best results in all three COVID-19 detection datasets, including 2-class and 3-class X-ray datasets. Additionally, ResNet152 with 2048 deep features gives good classification accuracies after the proposed FS approach. The 2-class and 3-class results on X-ray datasets, achieved by the deep features extracted by the ResNet18, are also very promising when compared to others. Although 1024 deep features of GoogLeNet also report impressive results for the COVID CT-Dataset, both VGG19 and VGG16 fail to produce promising results for all four datasets. In the case of VGG networks, a large number of features with a small number of training data, usually over-fit the ML classifiers. Accordingly, we can see a good margin in the classification accuracies as compared to other deep features. However, there is considerable evidence that the proposed approach of extracting combined global and local features from different layers of ResNet18 results in impressive outcomes over commonly used transfer learning-based deep feature extraction techniques.

**Figure 6:**
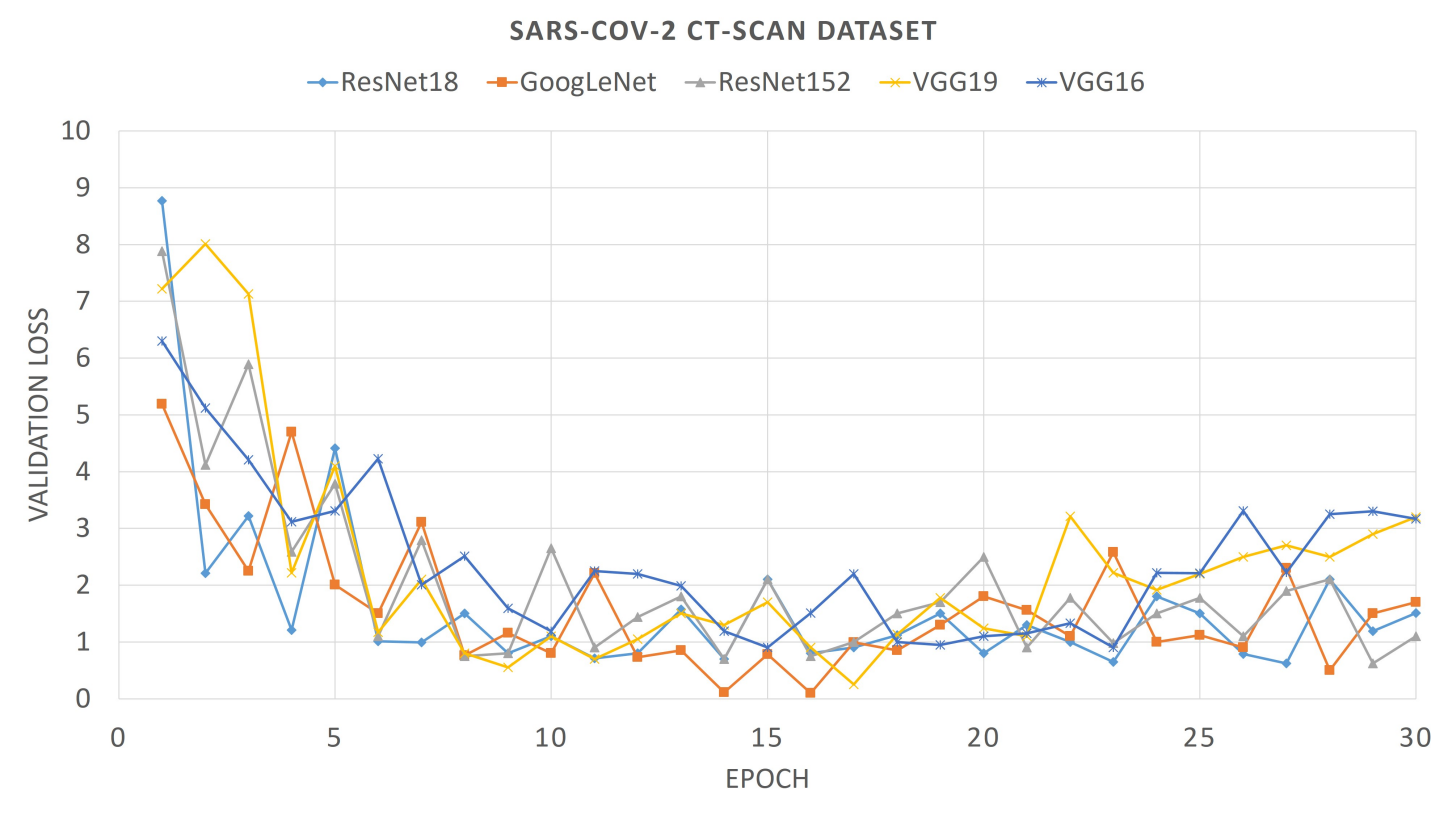
Loss plot of different pre-trained Convolutional Neural Networks (CNNs) on COVID CT-Dataset.

**Figure 7:**
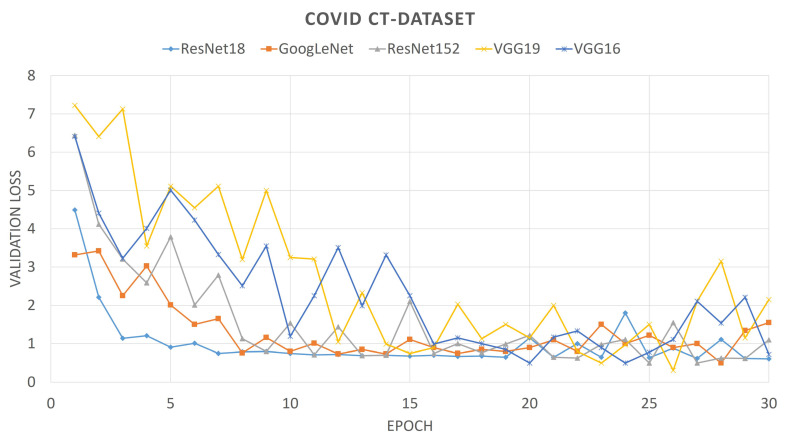
Loss plot of different pre-trained CNNs on SARS-Cov-2 dataset.

For comparison purposes, we also provide the convergence plots of validation losses and validation accuracies for 30 epochs of training, for each of the aforementioned deep CNNs. From Figure 6 and Figure 7, it can be seen that the validation loss plots of ResNet18 and GoogLeNet are somewhat stable and converge well, whereas the plots of VGG networks and ResNet152 are not that stable. It can also be observed that, for the SARS-Cov-2 CT-Scan dataset, the obtained loss plots are better than that of COVID CT-Dataset. These experimental phenomena can be described by considering the depths of the networks and the sizes of the datasets. The size of the COVID CT-Dataset is much less than that of the SARS-Cov-2 dataset, so deeper networks learn less and start overfitting in lesser epochs. For VGG networks, smaller datasets often cause gradient vanishing problem. For residual networks, like ResNet18, the skip-connections between intermediate layers address the problem of gradient vanishing. However, ResNet18 has a lesser number of layers than that of ResNet152, therefore it fits smaller datasets better. Accordingly, it is intuitive that ResNet18 learns better than any other CNN models considered here. This is observed from the validation accuracy plots of previously mentioned CNN models.

**Figure 8:**
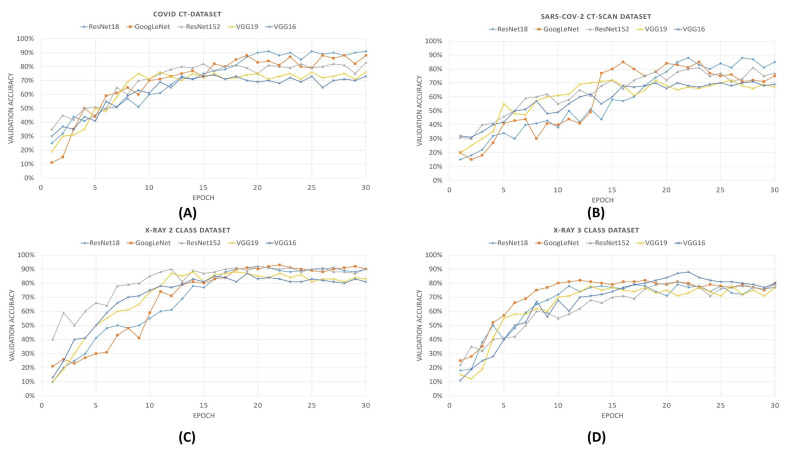
Validation accuracy plots of different pre-trained CNNs for all previously mentioned COVID-19 datasets. In the figure from (**A**–**D**) denotes the validation accuracy plots of COVID CT-Dataset, SARS-COV-2 CT-Scan Dataset, 2 calss and 3 class X-Ray dataset.

From the validation accuracy versus epoch plots that are shown in Figure 8, it is evident that ResNet18 achieves maximum accuracy among other deep neural networks. During training for 30 epochs, maximum accuracies are obtained by using ResNet18 for COVID CT-Dataset, SARS-Cov-2 CT-Scan datasets, and X-Ray 2 class dataset, but it takes more numbers of epochs to converge. For the 3-class dataset of X-ray, the accuracy obtained by ResNet152 is 82% which is more than that of ResNet18. The accuracies obtained by the ResNet18 and without optimization, in the COVID CT-Dataset and SARS-Cov-2 CT-Scan Dataset and 2-class and 3-class X-ray datasets, are 91%, 88%, 80%, and 92%, respectively.

Thus, we can conclude that ResNet18 learns more relevant and discriminatory deep features as compared to other CNNs with appropriate feature dimensions. Here, we have further optimized this feature vector using the proposed CGRO algorithm.

### 5.2. Classifier Selection for CGRO Algorithm

**Figure 9:**
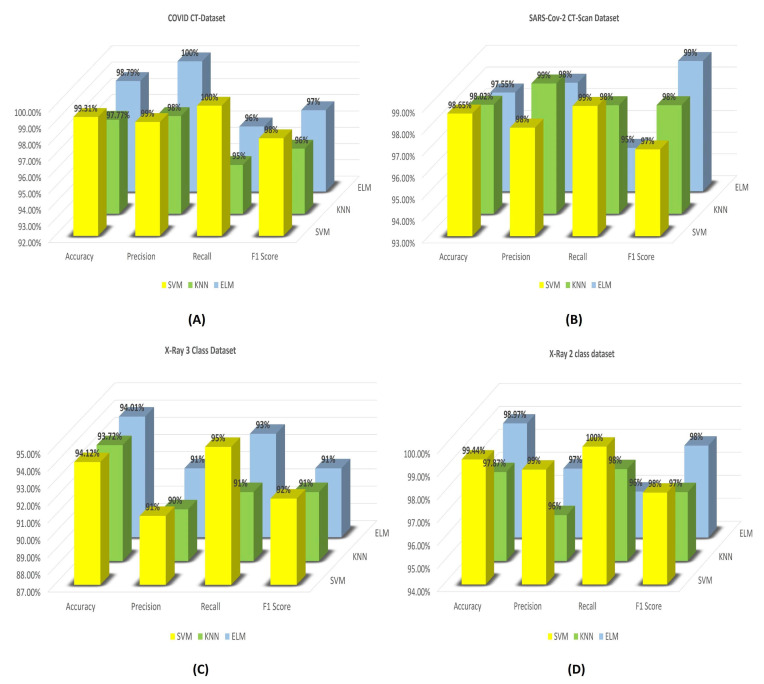
Results obtain by our proposed CGRO based FS algorithm using three different classifiers on: (**A**) COVID CT-Dataset, (**B**) SARS-Cov-2 CT-Scan dataset, (**C**) Chest X-ray 3-class of Muhammed Talo, and (**D**) Chest X-ray 2-class dataset of Muhammed Talo.

For the calculation of the fitness function of the GRO algorithm, we applied and implemented three popularly used ML classifiers, namely SVM, KNN, and ELM, for all three COVID-19 detection datasets. It is observed that the accuracies obtained with the SVM classifier are superior to the other two, as a whole. Whereas, if we consider all four primary measures of the evaluation, we see that the results obtained by the three classifiers are close to each other. The results obtained with these three classifiers on all the three COVID-19 datasets are shown by the bar charts that are given in Figure 9.

From the bar diagrams, it is seen that the binary classification accuracies that are obtained using the SVM classifier are the maximum accuracies amongst these all three. The SOTA accuracies achieved are 99.31%, 98.65%, 94.12%, and 99.44%. For SARS-Cov-2 CT-Scan Dataset, all three classifiers report similar classification accuracies with SVM yielding the highest among them. But it is seen that the performance of KNN is also impressive as it achieves the maximum recall of 99% and accuracy of 98.02% for this particular dataset. On the other hand, the maximum F1 score is obtained using the ELM classifier on SARS-Cov-2 CT-Scan Dataset. Whereas, for the COVID CT-Dataset, the SVM classifier consistently outperforms all other classifiers in terms of accuracy, recall, and F1 score. It is to be noted that the performance of the ELM classifier is also good for the COVID CT-Dataset. It achieves a maximum precision of 100% and reports 98.79% binary classification accuracy, which is much closer to that of SVM. Unlike the SARS-Cov-2 CT-Scan Dataset, in the COVID CT-Dataset, the KNN classifier fails to achieve good performance when compared to the other two classifiers. Similarly, for the 2-class and 3-class, the results obtained over the dataset of X-Ray with SVM and ELM classifiers are much similar, especially the classification accuracies. For the 3-class problem, the accuracies are 94.12%, 93.72%, and 94.01% obtained by SVM, KNN, and ELM classifiers, respectively, and it is seen that all of the accuracies are much closer to each other. Whereas, for the 2-class dataset, although the results of SVM and ELM classifiers are almost the same, the KNN classifier has failed to achieve good results. As a whole, it can be said that, even though the results obtained from different classifiers are almost comparable, SVM reports the best classification results. Therefore, in this study of Covid-19 detection, all other experimentations are done while using the SVM classifier with kernel function and regularization parameter fixed to ’rbf’ and 5000, respectively.

### 5.3. Hyperparameter Tuning

Hyperparameters always play an important role in boosting the results of the final model. Our entire ensemble work can be divided into some parts, which are the feature extraction using deep learning algorithms, feature optimization, and classification using traditional ML classifiers. Each section of this present ensemble learning has a specific set of hyperparameters that need to be optimized. To obtain a fixed set of optimal hyperparameters, we have performed exhaustive experimentation, which is discussed in this section.

In any deep learning framework, training, validation, and testing with appropriate hyperparameters have always been an important part of research studies. In this work, we have chosen the Adam optimizer for optimizing the cross-entropy loss, with a constant learning rate of $10^{-4}$, while keeping the momentum, step size, and other parameters fixed to their standard values.

**Figure 10:**
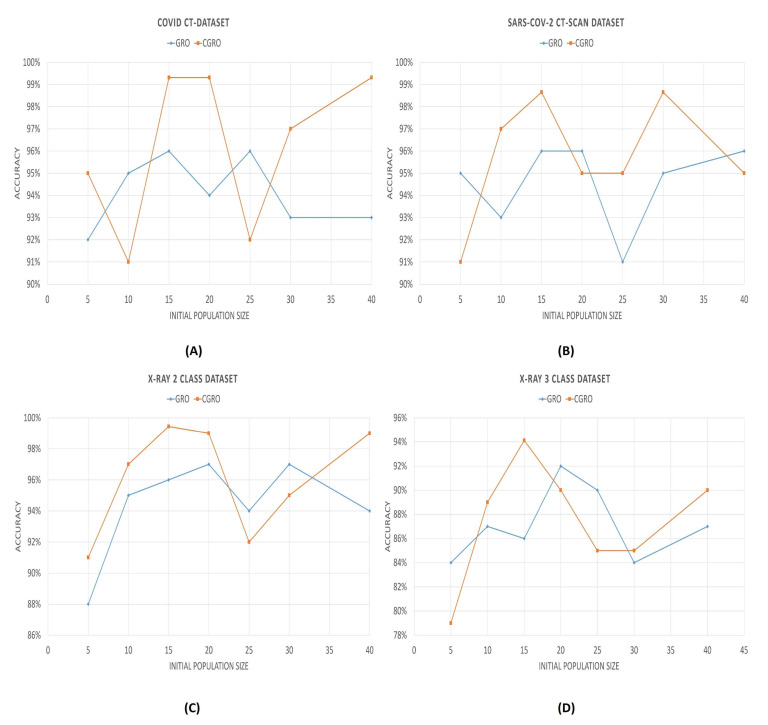
Graph showing the variation of classification accuracies with respect to the initial population size. In the Figure (**A**–**D**) denote the variations of COVID CT-Dataset, SARS-COV-2 CT-SCAN Dataset, 2 class and 3 class X-Ray dataset

**Figure 11:**
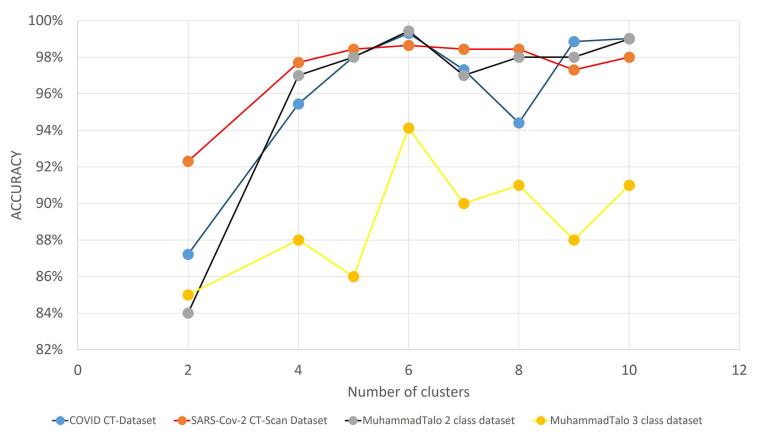
Graph showing the variation of classification accuracies with respect to the number of clusters for three COVID-19 datasets.

The wrapper-based FS algorithm also has some hyperparameters that significantly affect the performance of the model. Among all of these, the initial population size and number of clusters (in our case), play the most vital role in boosting the classification accuracy. Figure 10 and Figure 11 illlustrate the variation of the accuracies with varying initial population sizes and a varying number of clusters, respectively.

It can be observed from the population vs accuracy plots that the accuracies first reach the maximum value with the initial population of 15. The maximum is also hit again at different initial population sizes for different datasets, since a lesser initial population size implies lesser time consumption; therefore, we have fixed the population size of CGRO to 15. Howeve, the GRO algorithm does not follow such a fixed pattern, like CGRO. For the different datasets, the maximum accuracy is achieved with different initial population sizes.

The number of clusters is another important hyperparameter that has a significant contribution to achieve SOTA results in all three datasets. After doing several experimentations (the results reported in Figure 11), we have concluded that, for this present framework of optimizing deep features, the number of clusters for the proposed CGRO algorithm is set to 6. It is evident that, in our proposed framework of FS, the best classification performance is obtained with several clusters equal to 6.

### 5.4. Comparison With Other Popularly Used Algorithms

In order to estimate the performance of the CGRO algorithm, we have accumulated the results that were obtained by other popularly used wrapper type FS algorithms, such as Genetic Algorithm (GA) [62], Atom Search Optimizer (ASO) [63], Harmony Search Algorithm (HSA) [64], Particle Swarm Optimization (PSO) [65], and GRO itself without clustering-based population selection. The results are reported as a comparative study among these algorithms and the proposed CGRO algorithm in Table 6, Table 7, Table 8 and Table 9 for the COVID CT-Dataset, SARS-Cov-2 CT-Scan dataset, X-ray 2 class dataset, and X-ray 3 class dataset, respectively.

From the tables, it can be observed that the proposed CGRO algorithm achieves the best results among the previously mentioned FS algorithms. Even CGRO selects a very less number of feature vectors for an optimized solution vector. This fact of achieving impressive results with smaller feature space denotes the efficiency of the CGRO algorithm. Besides, the difference in accuracies and other evaluation parameters between GRO and CGRO algorithm is particularly noticeable; this implies that the clustering-based population improves the performance of the GRO algorithm with a significant margin of difference.

### 5.5. Comparison with SOTA Techniques

In this section, we report a comparative study of our proposed CGRO algorithm for deep features optimization, with other recently evolved models for COVID-19 detection on all three datasets.

Table 10, Table 11 and Table 12 report the study of comparison of CGRO based FS approach with some SOTA techniques for the COVID CT-Dataset, SARS-Cov-2 CT-Scan dataset, and Chest X-Ray dataset of Muhammed Talo, respectively. It is evident from Table 10, Table 11 and Table 12 that the proposed CGRO algorithm with deep residual features achieves SOTA results on all COVID CT-scan datasets, which are taken into account for the evaluation of our model, with a good margin of differences from other ML-based techniques, which have been developed for the detection COVID cases so far. It is observed that, on the COVID CT-Dataset, Saeedi et al. [31] extracted deep features of DenseNet121 and classified them using Nu-SVM classifier. The Nu-SVM is different from C-SVM in terms of the regularisation parameter. For C-SVM, regularization parameter C varies from zero to infinity, whereas, for Nu-SVM, the parameter ν varies between zero to one. The Nu-SVM gives a better estimation of the regularization than that of C-SVM. This ensemble of ML classifiers with deep features achieved 90.61% classification accuracy. Shaban et al. [32] implemented a purely traditional ML approach, which includes GLCM features, and these are optimized by hybrid FS model (HFSM) and classified using Enhanced KNN (EKNN) classifier. The author reported a very good classification accuracy of 96%. However, the classification results reported by the proposed CGRO algorithm are considerably superior to others. We reach a similar conclusion from Table 7 too but, for the SARS-Cov-2 CT-Scan dataset, the overall classification accuracies obtained by all other developed approaches are very promising. Soares et al. [20] proposed the dataset with the introduction of xDNN for classification purpose. Their proposed xDNN model achieves 97.38% accuracy. It is also observed that Jaiswal et al. [33] used traditional transfer learning with DenseNet201 and reported a 96.25% classification result. Although several approaches obtained impressive results on the SARS-Cov-2 CT-Scan dataset, our deep residual features with the CGRO algorithm model report best among them and achieve a SOTA accuracy of 98.65% for the binary class classification problem. The authors of the Covid-19 X-Ray dataset, Ozturk et al. [21], developed a completely new deep neural network, called DarkCovidNet, and achieved 87.02% (for 3 class) classification accuracy, which is much less than that of the accuracy achieved by our CGRO algorithm.

### 5.6. Statistical Significance Test: McNemar’s Test

The McNemar’s test is a non-parametric test for paired nominal data. We have performed the McNemar’s test to asses the statistical significance of the results obtained by the proposed algorithm. This test is performed by calculating McNemar’s parameter *X*, which can be calculated by the following equation.

(11) $$Z=\frac{\left(\right|a_{01}-a_{10}{\left|-1\right)}^{2}}{\left(a_{01}+a_{10}\right)}$$

In a statistical test, the process that shows that there is no difference between certain characteristics of population is known as a null hypothesis. In McNemar’s test, if the value of $Z_{i}>\chi_{\left(1,\alpha =0.05\right)}^{2}=3.84$, the degree of freedom is 1, and the statistical probability is 0.95, then the null hypothesis is rejected and the model has better performance than other models. The results of the McNemar’s test on three datasets are reported in Table 13. It is evident from the table that, for most of the cases, the null hypothesis is rejected, thereby concluding that our proposed algorithm can perform better than most of the existing algorithms.

## 6. Conclusions

In this paper, we have proposed a meta-heuristic FS method, named CGRO, based on Golden Ratio Optimizer with the clustering based population embedded in it. This FS method has been evaluated on three popular and publicly available X-ray and CT-scan images that are related to COVID-19, namely SARS-Cov-2, COVID-CT, and Chest X-Ray dataset. Our proposed method has achieved impressive classification accuracies of 98.65%, 99.31%, 99.44%, and 94.12%, respectively, on the said datasets. We compare the results obtained by the proposed CGRO algorithm with some popularly known optimization algorithms such as GA, HSA, ASO, PSO, and GRO. The results prove the superiority of the CGRO algorithm over those methods. Moreover, the research domain of COVID-19 has become a key interest in recent times, and various ML and deep learning-based models have been proposed by the researchers to recognize the COVID-19 patients automatically just by biomedical image processing (Chest X-ray and COVID-19). In our task, we have achieved SOTA results on three open-access datasets and obtained better results than some deep learning-based models. Even after achieving SOTA results, there are certain limitations of this framework, such as feature extraction itself is a lengthy procedure since features are extracted from deep learning models. In addition to this, feature selection is also a lengthy process as it requires several iterations to achieve the optimal set of feature vectors. Apart from that, this two-stage FS algorithm requires a feature set of promisingly large size. This is another limitation of this approach. Thereby, we conclude this paper with a small discussion of some future scopes, which are listed below.

- For feature extraction purpose, we have used some old pre-trained deep learning models; in recent times, lots of new deep learning nets have been developed for classification, such as capsule net, exception net, and so on. These can be used for deep feature extraction.
- Some ML classifiers have also been evolved in recent times. These classifiers can be used in calculating the fitness function of the CGRO algorithm and they can achieve better results.
- The CGRO algorithm can be hybridized with other FS algorithms, as proposed in recent times, which can improve the results with a good margin.

## Figures and Tables

**Table 1:** Features extracted from different layers of ResNet18 network.

Layer	Feature Map	Adaptive Average Pool	Feature Dimension	Number of Features
Layer1	64 × 56 × 56	YES	64 × 1 × 1	64
Layer2	128 × 28 × 28	YES	128 × 1 × 1	128
Layer3	256 × 14 × 14	YES	256 × 1 × 1	256
Layer4	512 × 7 × 7	YES	512 × 1 × 1	512
Dimension of the final feature vector				960

**Table 2:** The results obtained on Covid CT-Dataset using different models. Different parameters and obtained results with 4 layers features of ResNet18 are highlighted boldly in the table.

Deep Neural Network	No. of Features Obtained	No. of Features Selected by CGRO	Accuracy	Precision	Recall	F1 Score
**GoogLeNet**	1024	455	97.73%	92%	98%	95%
**VGG16**	25,088	12,345	91.27%	88%	90%	90%
**VGG19**	25,088	14,756	89.73%	91%	87%	88%
**ResNet152**	2048	1059	95.46%	91%	96%	97%
**ResNet18**	512	152	96.32%	97%	97%	94%
**From 4 layers of ResNet18**	**960**	**328**	**99.31%**	**99%**	**100%**	**98%**

**Table 3:** Results obtained on SARS-Cov-2 CT-scan dataset using different models. Different parameters and obtained results with 4 layers features of ResNet18 are highlighted boldly in the table.

Deep Neural Network	No. of Features Obtained	No. of Features Selected by CGRO	Accuracy	Precision	Recall	F1 Score
**GoogLeNet**	1024	312	96.15%	95%	98%	92%
**VGG16**	25,088	9472	87.99%	82%	89%	80%
**VGG19**	25,088	13,255	90.41%	87%	92%	87%
**ResNet152**	2048	944	96.77%	92%	92%	95%
**ResNet18**	512	301	95.41%	94%	95%	95%
**From 4 layers of ResNet18**	**960**	**252**	**98.65%**	**98%**	**99%**	**97%**

**Table 4:** Results obtained on X-Ray 2-class dataset using different models. Different parameters and obtained results with 4 layers features of ResNet18 are highlighted boldly in the table.

Deep Neural Network	No. of Features Obtained	No. of Features Selected by CGRO	Accuracy	Precision	Recall	F1 Score
**GoogLeNet**	1024	789	94.57%	91%	95%	95%
**VGG16**	25,088	16,789	90.11%	92%	90%	91%
**VGG19**	25,088	12,111	86.11%	88%	82%	84%
**ResNet152**	2048	1561	95.57%	93%	97%	92%
**ResNet18**	512	102	95.92%	94%	95%	94%
**From 4 layers of ResNet18**	**960**	**199**	**99.44%**	**99%**	**100%**	**98%**

**Table 5:** The results obtained on X-ray 3-class dataset using different models. Different parameters and obtained results with 4 layers features of ResNet18 are highlighted boldly in the table.

Deep Neural Network	No. of Features Obtained	No. of Features Selected by CGRO	Accuracy	Precision	Recall	F1 Score
**GoogLeNet**	1024	549	89.16%	90%	93%	88%
**VGG16**	25,088	11,259	83.54%	84%	81%	82%
**VGG19**	25,088	14,179	81.68%	80%	79%	84%
**ResNet152**	2048	1018	90.99%	93%	91%	88%
**ResNet18**	512	197	91.22%	92%	94%	93%
**From 4 layers of ResNet18**	**960**	**217**	**94.12%**	**91%**	**95%**	**92%**

**Table 6:** Comparative study of different optimization algorithms with proposed CGRO-based FS algorithm on the COVID CT-Dataset. The performance of proposed algorithm has been highlighted in bold text format.

Optimization Algorithm	No. of Features Selected	Accuracy	Precision	Recall	F1 Score
GA	412	95.53%	96%	93%	97%
HSA	332	94.17%	95%	96%	94%
ASO	557	96.44%	95%	97%	92%
PSO	225	95.13%	94%	95%	97%
GRO	397	97.77%	98%	99%	98%
**Proposed CGRO**	**328**	**99.31%**	**99%**	**100%**	**98%**

**Table 7:** Comparative study of different optimization algorithms with proposed CGRO based FS algorithm on SARS-Cov-2 CT-Scan dataset.The performance of proposed algorithm has been highlighted in bold text format.

Optimization Algorithm	No. of Features Selected	Accuracy	Precision	Recall	F1 Score
GA	502	91.65%	94%	87%	95%
HSA	211	92.17%	91%	93%	94%
ASO	444	94.41%	93%	95%	95%
PSO	345	96.98%	96%	95%	95%
GRO	311	95.13%	91%	93%	95%
**Proposed CGRO**	**252**	**98.65%**	**98%**	**99%**	**97%**

**Table 8:** Comparative study of different optimization algorithms with proposed CGRO based FS algorithm on Chest X-Ray 2 class dataset. The performance of proposed algorithm has been highlighted in bold text format.

Optimization Algorithm	No. of Features Selected	Accuracy	Precision	Recall	F1 Score
**GA**	426	90.13%	88%	90%	91%
**HSA**	357	93.29%	91%	94%	93%
**ASO**	229	97.44%	95%	98%	96%
**PSO**	513	95.39%	92%	97%	97%
**GRO**	643	96.92%	95%	93%	96%
**Proposed CGRO**	**199**	**99.44%**	**99%**	**100%**	**98%**

**Table 9:** Comparative study of different optimization algorithms with proposed CGRO based FS algorithm on Chest X-Ray 3 class dataset. The performance of proposed algorithm has been highlighted in bold text format.

Optimization Algorithm	No. of Features Selected	Accuracy	Precision	Recall	F1 Score
**GA**	491	85.13%	87%	84%	83%
**HSA**	231	81.11%	79%	82%	82%
**ASO**	497	90.77%	92%	91%	91%
**PSO**	319	89.77%	92%	91%	90%
**GRO**	412	92.19%	93%	90%	94%
**Proposed CGRO**	**217**	**94.12%**	**91%**	**95%**	**92%**

**Table 10:** Performance comparison of our proposed approach with some existing works for COVID CT-Dataset. Maximum value is bolded.

Work Reference	Feature	Method of Classification	Accuracy
Loey et al. [30]	Deep features	Data augmentation with classical augmentation technique and CGAN	82.91%
Jhao et al. [19]	Pre-trained CNN learns by itself	TL by DenseNet161 + CSSL	89.1%
Saeedi et al. [31]	Deep features of DenseNet121	Nu-SVM	90.61% ± 5%
Sakagianni et al. [66]	NA	AutoML Cloud Version	88.31%
Shaban et al. [32]	GLCM	HFSM and EKNN classifier	96%
Proposed method	Deep features of ResNet18	FS and classification using CGRO algorithm	**99.31%**

**Table 11:** Performance comparison of our proposed approach with some existing works for SARS-Cov-2 CT-Scan dataset. Maximum value is bolded.

Work Reference	Feature	Method of Classification	Accuracy
Jaiswal et al. [33]	Deep neural network learns relevant features by itself	DenseNet201	96.25%
Soares et al. [20]	Automated classification with deep xDNN	xDNN	97.38%
Soares et al. [20]	Ensemble learning and classification	Adaboost	95.16%
Panwar et al. [34]	Deep neural architecture	Grad-CAM	95.61%
Proposed method	Deep features of ResNet18	FS and classification using CGRO algorithm	**98.65%**

**Table 12:** Performance comparison of our proposed approach with some existing works for Chest X-Ray dataset of Muhammed Talo. Maximum value is bolded.

Work Reference	Feature	Method of Classification	Accuracy
Ozturk et al. [21]	No traditional features were extracted, end-to-end deep neural network is proposed	DarkCovidNet	2-Class : 98.08% 3-Class: 87.02%
Abdulrahaman et al. [35]	Deep features of hidden and visible layers	Deep belief network	3-Class: 90%
Proposed method	Deep features of ResNet18	FS and classification using CGRO algorithm	**2-Class: 99.44**% **3-Class: 94.12**%

**Table 13:** Statistical comparison using McNemar’s test of our proposed CGRO algorithm with some existing SER methods.

COVID-CT Dataset					SARS-Cov-2 Dataset					X-ray Dataset of Muhammed Talo				
**Competitor Algorithms (B)**	**Control Algorithm (A)**				**Competitor Algorithms (B)**	**Control Algorithm (A)**				**Competitor Algorithms (B)**	**Control Algorithm (A)**			
	$a_{01}$	$a_{10}$	$Z_{i}$	**Status of Null Hypothesis**		$a_{01}$	$a_{10}$	$Z_{i}$	**Status of Null Hypothesis**		$a_{01}$	$a_{10}$	$Z_{i}$	**Status of Null Hypothesis**
Loey et al.	1	9	4.9	Reject	Jaiswal et al.	21	71	26.1	Reject	Ozturk et al.	10	55	29.78	Reject
Jhao et al.	2	12	5.78	Reject	Soares et al.	10	66	39.8	Reject	Abdulrahaman et al.	8	20	0.39	Accept
Saeedi et al.	1	10	5.81	Reject	Soares et al.	11	50	23.67	Reject	NA	NA	NA	NA	NA
sakagianni et al.	4	20	9.37	Reject	Panwar et al.	30	89	28.26	Reject	NA	NA	NA	NA	NA
Shaban et al.	3	7	0.9	Accept	NA	NA	NA	NA	NA	NA	NA	NA	NA	NA

## Data Availability

No new data were created or analyzed in this study. Data sharing is not applicable to this article.
